# Tocilizumab in refractory pretibial myxedema: new therapeutic insights

**DOI:** 10.1210/jcemcr/luag249

**Published:** 2026-10-22

**Authors:** Silvia Corrias, Aurora Arba, Filippo Lixi, Giuseppe Giannaccare, Francesco Boi, Giulia Lanzolla

**Affiliations:** Endocrinology Unit, Department of Medical Sciences and Public Health, University of Cagliari, 09042 Monserrato, CA, Italy; Endocrinology Unit, Department of Medical Sciences and Public Health, University of Cagliari, 09042 Monserrato, CA, Italy; Ophthalmology Unit, Department of Surgical Sciences, University of Cagliari, 09042 Monserrato, CA, Italy; Ophthalmology Unit, Department of Surgical Sciences, University of Cagliari, 09042 Monserrato, CA, Italy; Endocrinology Unit, Department of Medical Sciences and Public Health, University of Cagliari, 09042 Monserrato, CA, Italy; Endocrinology Unit, Department of Medical Sciences and Public Health, University of Cagliari, 09042 Monserrato, CA, Italy

**Keywords:** pretibial myxedema, thyroid dermopathy, Graves orbitopathy, thyroid eye disease, tocilizumab, interleukin-6 receptor

## Abstract

Pretibial myxedema (PTM) is a rare extrathyroidal manifestation of Graves disease, often associated with Graves orbitopathy (GO). Tocilizumab, an interleukin-6 receptor inhibitor, is effective in refractory GO, but its role in PTM is unclear. We report the case of a 72-year-old woman with longstanding Graves disease who developed glucocorticoid-refractory GO and diffuse glucocorticoid-refractory PTM. Tocilizumab (8 mg/kg intravenously every 4 weeks) was initiated as second-line treatment of moderate to severe active GO. After 6 infusions, the patient experienced a significant improvement in both activity and severity of GO, while PTM showed near-complete resolution of edema and skin induration. No adverse events were recorded. Our findings suggest that interleukin-6 receptor inhibition with tocilizumab may represent a novel therapeutic option for refractory thyroid dermopathy, particularly in the setting of concomitant GO.

## Introduction

Pretibial mixedema (PTM), also known as thyroid dermopathy, is a rare extrathyroidal manifestation of Graves disease (GD), occurring in ∼4% of patients with Graves orbitopathy (GO) [1, 2]. Clinically, PTM presents as nonpitting edema with well-defined margins, often with indurated erythematous plaques exhibiting a peau d’orange appearance and, in advanced stages, nodular lesions [2]. The pretibial region is predominantly affected, although involvement of the feet, toes, upper limbs, and shoulders has also been reported [2]. Histopathologically, PTM is characterized by the presence of few lymphocytes, a moderate increase of mast cells, and the dermal accumulation of glycosaminoglycan and mucin [2]. Glycosaminoglycan and mucin deposit promote fluid retention, skin thickening, and, in severe cases, lymphatic compression and occlusion, with subsequent lymphedema or elephantiasis [3]. While the pathogenesis of PTM remains incompletely understood, it is thought to share key mechanisms with GO. However, this analogy is largely inferential given the paucity of dermopathy-specific research. The prevailing hypothesis implicates thyrotropin receptor antibody (TRAb)-mediated activation of thyroid-stimulating hormone (TSH) receptors on dermal fibroblasts. This is supported by the demonstrated presence of TSH receptor transcripts in cultured skin fibroblasts [4] and the high TRAb titers in patients with PTM [5]. Autoimmunity against the insulin-like growth factor 1 receptor may further contribute. As in GO, in addition to T and B lymphocyte, pro-inflammatory cytokines including interleukin-1β (IL-1β), interleukin-6 (IL-6), tumor necrosis factor-α, transforming growth factor-β, and interferon-γ have a key role in sustaining the autoimmune reaction [6]. Diagnosis is primarily clinical, with skin biopsy reserved for uncertain cases [2].

Treatment of PTM remains primarily symptomatic, based on topical corticosteroids, preferably under occlusive dressing in early stages, while intralesional corticosteroids may have a role in plaque and nodular forms [1, 2]. Tocilizumab, an IL-6 receptor antagonist recommended as second-line therapy for glucocorticoid (GC)-refractory GO [7], has not been explored in PTM, and no clinical data are currently available. Here, we describe the first reported case of GC-refractory PTM successfully treated with tocilizumab.

## Case presentation

We report the case of a 72-year-old female patient with GD diagnosed in 2017, initially managed with antithyroid drugs for 12 months, followed by total thyroidectomy in 2022, with histological diagnosis of macrofollicular adenomatous goiter. She has been maintained on levothyroxine replacement therapy since.

Approximately 3 years after GD diagnosis, she developed moderate to severe active GO and received high-dose intravenous GCs (methylprednisolone 500 mg × 6 weekly cycles, then 250 mg × 6 cycles) without clinically meaningful improvement. Concurrent bilateral PTM was treated with topical corticosteroids under occlusion for several years, with limited benefit.

The patient had no other current or past dermatologic conditions and no clinical evidence suggestive of other autoimmune diseases. Therefore, a comprehensive organ-specific autoantibody panel was not performed, and the presence of subclinical autoimmunity cannot be entirely excluded. Family history was notable for autoimmune thyroid disease, with a sister affected by chronic autoimmune thyroiditis.

## Diagnostic assessment

The patient was referred to our center in March 2025 because of GO worsening. She presented with ocular irritative symptoms and intermittent diplopia. Moreover, she reported a severe worsening of PTM over the past year, with markedly thickened, indurated, warm, and erythematous skin, difficulty wearing tight trousers, and diffuse pain in the pretibial region. Under levothyroxine 88 μg/day, laboratory testing showed TSH 0.05 mU/L (reference range, 0.55-4.78), free thyroxine 1.5 ng/dL (International System of Units [SI]: 19.3 pmol/L; reference range, 0.89-1.76 ng/dL [9-23 pmol/L]), free triiodothyronine 3.2 pg/mL (SI: 4.91 pmol/L; reference range, 2.3-4.2 mg/dL [SI: 3.5-6.5 pmol/L]), and TRAb 40 IU/L (reference range, <1.75). Levothyroxine dosage was reduced to 75 μg/day. Ophthalmological examination showed bilateral proptosis of 25 mm, measured by Hertel exophthalmometer, eyelid width of 16 mm, and a clinical activity score (CAS) of 5/7 (eyelid edema, eyelid hyperemia, conjunctival hyperemia, conjunctival edema, and caruncular swelling), with the right eye being predominantly affected (Table 1). No lagophthalmos was observed. Inconstant diplopia was detected by Gorman score due to ocular motility restriction in upgaze and adduction. Dermatological examination confirmed bilateral diffuse infiltrative plaque-type PTM, with nonpitting edema, skin thickening, and a peau d’orange appearance (Fig. 1A).

**Figure 1:**
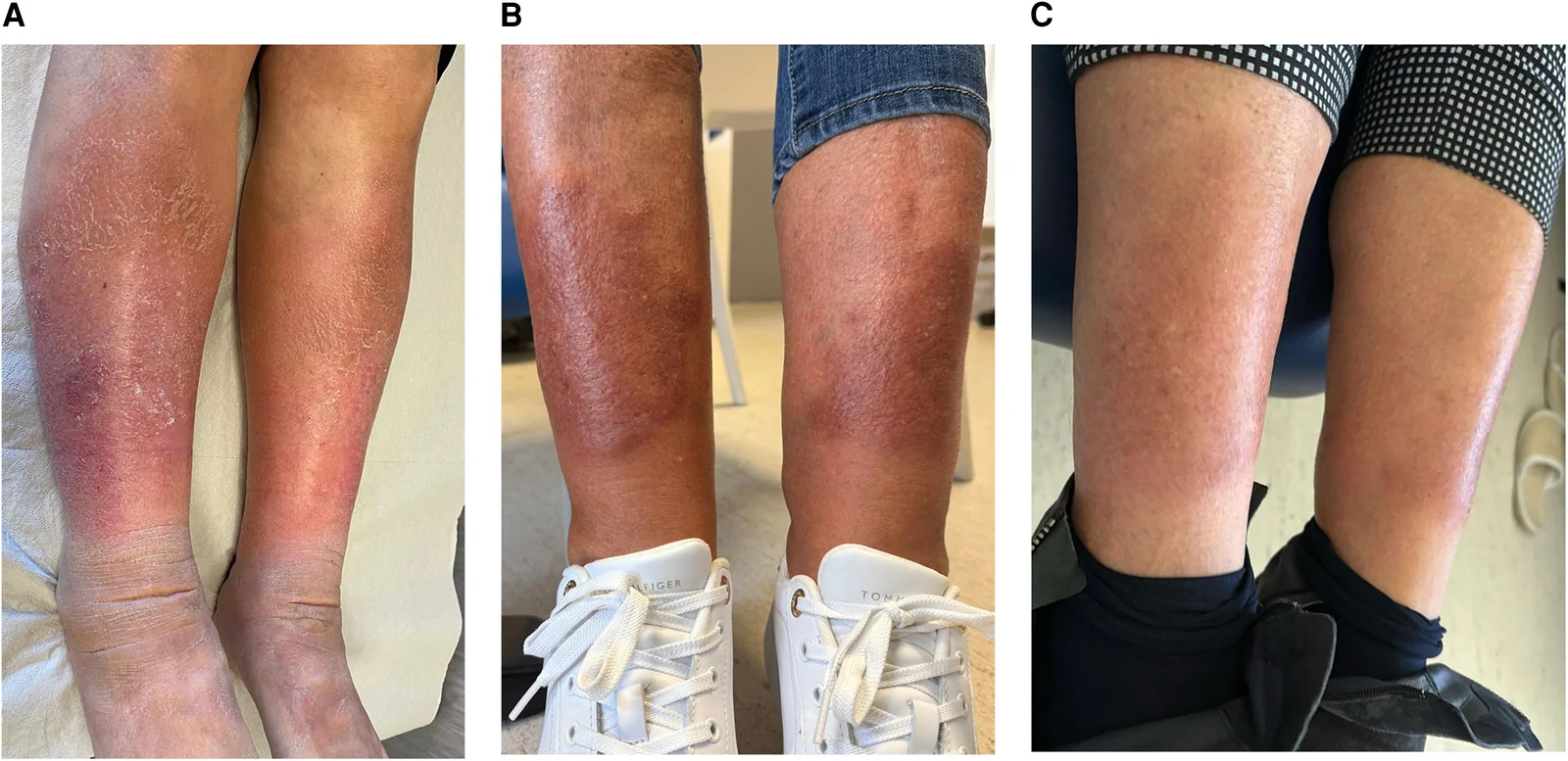
Improvement of PTM after treatment with tocilizumab. Clinical appearance of bilateral pretibial myxedema before starting therapy (A), after the second infusion of tocilizumab (B), and after completion of treatment (Day 180) (C).

**Table 1:** Ophthalmological and dermatological findings at baseline, after completion of tocilizumab treatment (Day 180), and at 6-month posttreatment follow-up (Day 360)

	Baseline		Post-TCZ (Day 180)		Post-TCZ (Day 360)	
Finding	RE	LE	RE	LE	RE	LE
Proptosis (mm)	25	25	23	22	21	20
Eyelid width (mm)	16	16	13	13	12	12
Eyelid edema	Moderate	Moderate	Mild	Absent	Absent	Absent
Eyelid hyperemia	Mild	Mild	Absent	Absent	Absent	Absent
Lagophthalmos	Absent	Absent	Absent	Absent	Absent	Absent
Conjunctival hyperemia	Marked	Moderate	Mild	Absent	Absent	Absent
Chemosis	Moderate	Mild	Absent	Absent	Absent	Absent
Caruncular edema	Moderate	Mild	Absent	Absent	Absent	Absent
Spontaneous orbital pain	Absent	Absent	Absent	Absent	Absent	Absent
Orbital pain due to eye movement	Absent	Absent	Absent	Absent	Absent	Absent
CAS (points)	5/7	5/7	2/7	0/7	0/7	0/7
Diplopia	Inconstant		Inconstant		Intermittent	
PTM	Diffuse infiltrative plaques, nonpitting edema, and peau d'orange		Near-complete resolution of edema and induration			

Abbreviations: CAS, Clinical Activity Score (7-item scale); LE, left eye; PTM, pretibial myxedema; RE, right eye; TCZ, tocilizumab.

## Treatment

Given moderate to severe and active GO unresponsive to first-line therapy, second-line treatment with tocilizumab was initiated at 8 mg/kg intravenously every 4 weeks for a total of 6 cycles. Following the second infusion, a significant improvement in GO was documented. Notably, a rapid and unexpected improvement in PTM was observed, with reduction of edema and skin induration (Fig. 1B). Subsequent evaluations confirmed a progressive decrease in dermal infiltration, attenuation of the peau d’orange appearance, and reduced skin thickness on palpation, along with concomitant improvement in erythema, pain, and local warmth.

## Outcome and follow-up

After completion of 6 infusions, GO was significantly improved compared with baseline findings. Proptosis decreased to 23 mm in the right eye and 22 mm in the left eye, with eyelid width reduced to 13 mm in both eyes, and CAS improved from 5/7 to 2/7 (Table 1). The patient no longer experienced constant diplopia but reported intermittent diplopia. Similarly, PTM showed a marked overall improvement with near-complete resolution of edema, skin induration, and peau d’orange appearance (Fig. 1C). Thyrotropin receptor antibody serum levels remained persistently elevated (35 IU/L). No clinically relevant adverse events were observed.

At follow-up visits conducted 3 and 6 months after the last tocilizumab infusion, the patient demonstrated sustained and progressive improvement of GO, with further reduction of periorbital soft tissue changes compared with end-of-treatment assessment, and CAS 0/7. Pretibial myxedema remained stable, with maintained near-complete resolution of edema and skin induration observed at treatment completion. No relapse of either GO or PTM was documented throughout the follow-up period. The patient reported no adverse events, and her general condition remained good.

## Discussion

Tocilizumab is a monoclonal antibody targeting the IL-6 receptor, approved for several inflammatory diseases and recommended by EUGOGO 2021 guidelines [7] as a second-line treatment for moderate to severe active GO unresponsive to first-line high-dose intravenous GC therapy. Interleukin-6 is a pleiotropic pro-inflammatory cytokine produced by fibroblasts, T and B lymphocytes, as well as antigen-presenting cells. It plays a key role in immune modulation by promoting T-cell proliferation and B-cell differentiation, thereby sustaining the autoimmune response in GO and PTM [6, 8, 9]. Indeed, IL-6 is overexpressed in orbital fibroblasts [9, 10] and promotes TSH receptor upregulation on orbital preadipocyte fibroblasts, providing a mechanistic basis for IL-6 receptor blockade in GO. In a small placebo-controlled randomized clinical trial enrolling 32 patients with GC-resistant GO, intravenous tocilizumab (8 mg/kg every 4 weeks for 4 cycles) achieved significantly higher rates of disease inactivation compared with placebo, although a notable inactivation rate was also observed in the placebo arm, possibly reflecting delayed effects of prior GC therapy or spontaneous disease remission [11]. Findings from additional uncontrolled and retrospective series support its efficacy in GC-resistant disease, although larger multicenter randomized clinical trials are needed.

Dermal fibroblasts secrete IL-6 in response to injury, but it is also constitutively overproduced in systemic sclerosis, contributing to skin fibrosis. Indeed, IL-6 acts on fibroblasts to drive collagen production and stimulate cell migration [12]. Although the exact role of IL-6 in PTM is not characterized, the pathophysiologic features of PTM are thought to be like that of GO. Thus, in theory, tocilizumab's mechanism of action in PTM is likely similar to what has been observed in GO. This hypothesis is supported by the knowledge that other biological agents commonly used for GO treatment, such as rituximab [13, 14] and teprotumumab [15-20], have been reported to be effective in PTM. Indeed, Smith et al reported that immunomodulatory therapy with rituximab and intravenous immunoglobulin in 8 patients with refractory PTM led to clinical improvement or stabilization in 75% of cases (80% with rituximab and 66% with intravenous immunoglobulin), supporting a potential role for these agents in refractory disease [13]. More recently, teprotumumab has been shown to improve PTM in patients with GO, although evidence remains limited, and adverse effects, including hearing impairment, have been described [15-20].

In our patient, treatment with tocilizumab was associated with a rapid and sustained improvement in PTM, already evident after the second infusion and progressively confirmed during follow-up. This early response may support a direct effect of IL-6 blockade on dermal fibroblast activity and glycosaminoglycan deposition. Notably, the parallel improvement of both PTM and GO further supports the concept of a shared pathogenic pathway underlying these GD extrathyroidal manifestations. The persistence of elevated TRAb serum levels suggests that the disease clinical course may not be solely dependent on circulating autoantibody titers but rather on downstream inflammatory and fibroblast-mediated mechanisms. This observation may have therapeutic implications, highlighting the potential value of targeting cytokine pathways independently of antibody levels.

Our findings support the hypothesis that IL-6 inhibition with tocilizumab represents a promising therapeutic strategy not only for GC-refractory GO but also for thyroid dermopathy. The rapid and sustained clinical improvement observed in both cutaneous and orbital manifestations highlights the potential of targeted cytokine inhibition as a novel approach in this setting. Larger prospective studies are warranted to confirm these observations and better define efficacy, safety, and optimal management of tocilizumab for PTM treatment.

## Learning points

- Tocilizumab, an IL-6 receptor inhibitor used as second-line therapy for GC-refractory GO, may also represent a novel therapeutic option for refractory PTM, suggesting a shared cytokine-driven pathogenic pathway between these extrathyroidal manifestations of GD.
- In patients with concomitant active GO and PTM refractory to corticosteroids, tocilizumab treatment may lead to rapid and sustained improvement of both conditions, supporting the concept of overlapping immunopathogenic mechanisms involving dermal and orbital fibroblast activation.
- Clinical improvement of PTM under tocilizumab may occur independently of circulating TRAb levels, suggesting that downstream inflammatory and fibroblast-mediated mechanisms rather than autoantibody titers alone may drive disease activity and therapeutic response.
- Interleukin-6 blockade may exert a direct effect on dermal fibroblast activity and glycosaminoglycan deposition, providing a mechanistic rationale for its use in thyroid dermopathy analogous to its established role in GO.
- Alongside rituximab and teprotumumab, tocilizumab may expand the therapeutic options for refractory thyroid dermopathy, offering a targeted cytokine-based alternative in patients with insufficient response to conventional topical and systemic corticosteroid therapy.

## Contributors

All authors made individual contributions to authorship. S.C. and G.L. were involved in the diagnosis and management of the patient. F.L. and G.G. were involved in the ophthalmological evaluation of the patient. S.C. and A.A wrote the original draft of the manuscript. A.A. acquired pictures of the patient and contribute to the conception of the manuscript. G.L. wrote the final version of the manuscript. F.B. provided intellectual contribution to the analysis and interpretation of the case and critically revised the manuscript. All authors reviewed and approved the final draft.

## Data Availability

Original data generated and analyzed during this study are included in this published article.
